# Translating the BDI and BDI-II into the HAMD and vice versa with equipercentile linking

**DOI:** 10.1017/S2045796019000088

**Published:** 2019-03-14

**Authors:** Toshi A. Furukawa, Mirjam Reijnders, Sanae Kishimoto, Masatsugu Sakata, Robert J. DeRubeis, Sona Dimidjian, David J.A. Dozois, Ulrich Hegerl, Steven D. Hollon, Robin B. Jarrett, François Lespérance, Zindel V. Segal, David C. Mohr, Anne D. Simons, Lena C. Quilty, Charles F. Reynolds, Claudio Gentili, Stefan Leucht, Rolf R. Engel, Pim Cuijpers

**Affiliations:** 1 Departments of Health Promotion and Human Behavior and of Clinical Epidemiology, Kyoto University Graduate School of Medicine/School of Public Health, Kyoto, Japan; 2 Department of Clinical, Neuro-, and Developmental Psychology, Amsterdam Public Health Research Institute, Vrije Universiteit Amsterdam, The Netherlands; 3 Department of Health Promotion and Human Behavior, Kyoto University Graduate School of Medicine/School of Public Health, Kyoto, Japan; 4 Department of Psychology, University of Pennsylvania, Philadelphia, PA, USA; 5 Department of Psychology and Neuroscience, University of Colorado, Boulder, USA; 6 Department of Psychology, University of Western Ontario, Westminster Hall, London, Ontario N6A 3K7, Canada; 7 Department of Psychiatry and Psychotherapy, University of Leipzig, Leipzig, Germany; 8 Department of Psychology, Vanderbilt University, Nashville, TN, USA; 9 Department of Psychiatry, The University of Texas Southwestern Medical Center, Dallas, TX, USA; 10 Department of Psychiatry and Addiction, Université de Montréal, Montréal, Québec, Canada; 11 Department of Psychology, University of Toronto – Scarborough, Toronto, Canada; 12 Center for Behavioral Intervention Technologies, Feinberg School of Medicine, Northwestern University, Chicago, IL, USA; 13 Department of Psychology, University of Oregon, Eugene, OR, USA; 14 Campbell Family Mental Health Research Institute, Centre for Addiction and Mental Health; Department of Psychiatry, University of Toronto, Toronto, Canada; 15 Department of Psychiatry, University of Pittsburgh Medical Center, Western Psychiatric Institute and Clinic, Pittsburgh, USA; 16 Department of General Psychology, University of Padova, Padova, Italy; 17 Department of Psychiatry and Psychotherapy, Technische Universität München, Kkinikum rechts der Isar, Germany; 18 Department of Psychiatry and Psychotherapy, Ludwig-Maximillians Universität München, Germany

**Keywords:** Assessment, Beck Depression Inventory, depressive disorder, Hamilton Rating Scale for Depression, rating instrument

## Abstract

**Aims:**

The Hamilton Depression Rating Scale (HAMD) and the Beck Depression Inventory (BDI) are the most frequently used observer-rated and self-report scales of depression, respectively. It is important to know what a given total score or a change score from baseline on one scale means in relation to the other scale.

**Methods:**

We obtained individual participant data from the randomised controlled trials of psychological and pharmacological treatments for major depressive disorders. We then identified corresponding scores of the HAMD and the BDI (369 patients from seven trials) or the BDI-II (683 patients from another seven trials) using the equipercentile linking method.

**Results:**

The HAMD total scores of 10, 20 and 30 corresponded approximately with the BDI scores of 10, 27 and 42 or with the BDI-II scores of 13, 32 and 50. The HAMD change scores of −20 and −10 with the BDI of −29 and −15 and with the BDI-II of −35 and −16.

**Conclusions:**

The results can help clinicians interpret the HAMD or BDI scores of their patients in a more versatile manner and also help clinicians and researchers evaluate such scores reported in the literature or the database, when scores on only one of these scales are provided. We present a conversion table for future research.

## Introduction

It is important to evaluate the course of major depressive disorder (MDD) using quantitative rating scales of symptoms. Various rating scales have been developed to evaluate the severity of MDD in research and clinical settings. These measures can be categorised as clinician-rated scales such as the Hamilton Rating Scale for Depression (HAMD) (Hamilton, 1960; Williams *et al*., 2008), Montgomery Åsberg Depression Rating Scale (MADRS) (Montgomery and Asberg, 1979) or Quick Inventory of Depression Symptomatology Clinician Rating (Rush *et al*., 2003), and self-report scales such as the Beck Depression Inventory (BDI) (Beck *et al*., 1961) and its revised version (BDI-II) (Beck *et al*., 1996), Patient Health Questionnaire-9 (Kroenke *et al*., 2001) or Quick Inventory of Depression Symptomatology self-report version (Rush *et al*., 2003). Although numerous scales for rating depression severity have been developed to date, the HAMD is the most commonly used clinician-rated scale in research and clinical settings. The HAMD has been used as a main outcome measure in randomised controlled trials of pharmacotherapy and psychotherapy for depression. In the latest network meta-analysis of antidepressant medications for MDD, 464 of 522 eligible studies reported baseline severity scores using the HAMD (Cipriani *et al*., 2018). Similarly, the network meta-analysis of psychotherapy for MDD showed that 75 of 198 studies reported outcomes using the HAMD (Barth *et al*., 2013). On the other hand, the BDI is one of the most widely used self-rating scales. The BDI/BDI-II have been used particularly often as the outcome measure in psychotherapy trials. According to the above-mentioned network meta-analysis studies of psychotherapies for depression, 116 of 198 studies used the BDI and 25 of 198 studies used the BDI-II as an outcome measure of the trial (Barth *et al*., 2013).

Although both the HAMD and the BDI/BDI-II are standard measures to assess depression severity, no study has yet examined how scores on the HAMD can be converted to the BDI/BDI-II scores or vice versa. It is important to link these two most commonly used scales for comparison of the baseline severity or treatment outcome. Several studies identified the corresponding scores of simultaneous HAMD and other scales such as MADRS (Leucht *et al*., 2018) and the Clinical Global Impression (Leucht *et al*., 2013*a*) using the equipercentile linking method (Linn, 1993). The equipercentile linking method has been used extensively for various other scales in previous publications (Leucht *et al*., 2005, 2006, 2013*b*, 2016; Furukawa *et al*., 2009; Levine and Leucht, 2013; Samara *et al*., 2014). In the current study, we attempted to link the HAMD and the BDI/BDI-II applying the same procedure.

## Method

### Database

We used an existing database of psychological treatments for depression which is updated annually through comprehensive literature searches in the bibliographic databases of PubMed, PsycINFO, EMBASE and the Cochrane Library (Cuijpers *et al*., 2008). Appendix A provides the full search strings used. This database has been used in a series of previously published meta-analyses (Bower *et al*., 2013; Furukawa *et al*., 2017; Karyotaki *et al*., 2017). For this linking study, we focused on the individual participant data (IPD) that we had assembled to conduct IPD meta-analytic studies comparing cognitive-behavioural therapy (CBT), antidepressant pharmacotherapy and their combination (Weitz *et al*., 2017).

### Rating scales

The HAMD is based on clinical interviews. We used the HAMD 17-item version in this analysis. The 17 items consists of nine symptoms (depressed mood, self-depreciation and guilt feelings, suicidal impulses, work and interests, psychomotor retardation, agitation, anxiety psychic, anxiety somatic, hypochondriasis) rated between 0 (absent) to 4 (very severe), and eight symptoms (initial insomnia, middle insomnia, delayed insomnia, gastrointestinal, general somatic, sexual interests, loss of insight, weight loss) rated between 0 (absent) to 2 (clearly present) (Hamilton, 1960). The maximum score of the HAMD is therefore 52. A meta-analysis showed that the HAMD has sufficient internal consistency (Cronbach's *α* = 0.79), inter-rater reliability (intra-class correlation coefficient (ICC) = 0.94) and test–retest reliability (ICC = 0.93) (Trajkovic *et al*., 2011).

The BDI is a 21-item patient's self-report questionnaire that measures the depression severity (Beck *et al*., 1961). All items of the BDI are rated on a four-point Likert scale ranging from 0 to 3, and the total score therefore ranges from 0 to 63. Beck *et al*. developed the revised version of the BDI to harmonise its item contents with the modern diagnostic criteria for MDD in Diagnostic and Statistical Manual of Mental Disorders (DSM)-IV, while maintaining the same number of items and range of scale as the BDI (Beck *et al*., 1996). The BDI has sufficient internal consistency in psychiatric patients (Cronbach's *α* ranging from 0.76 to 0.95) and non-psychiatric populations (Cronbach's *α* ranging from 0 .73 to 0.92) (Beck *et al*., 1988). The BDI-II also has sufficient internal consistency (*α* = 0.93 among college students, *α* = 0.92 among outpatients) (Beck *et al*., 1996). According to a survey of 1022 undergraduate students, the mean score of the BDI-II was 1.54 points higher than that of the BDI (Dozois *et al*., 1998). However, the two scales showed high correlation (*r* = 0.93), suggesting convergence of the two scales.

### Statistical analysis

We first drew scatterplots and calculated Spearman correlation coefficients between HAMD and BDI or BDI-II, at baseline and at end of treatment. We then applied the equipercentile linking procedure (Linn, 1993), which is a technique that identifies those scores on the HAMD and the BDI or the BDI-II that have the same percentile ranks, thus allowing for a nominal translation from HAMD scores to BDI or BDI-II scores or vice versa by using their percentile values. We used Microsoft Excel® to realise the analytical procedures described in Chapter 2 of Kolen and Brennan (1995) and to draw the diagrams. We merged the baseline and endpoint measurements to produce the final linking curves and the table of conversion.

Because many trials take the change scores from baseline to end of treatment, instead of raw scores at end of treatment, as the primary outcome, we also examined the linking relationships between change scores of the HAMD and the BDI/BDI-II.

### Subgroup and sensitivity analyses

In order to examine possible subgroup differences, we conducted the same analyses for men and women separately, and also for dropouts.

## Results

### Included studies

**Fig. 1.**
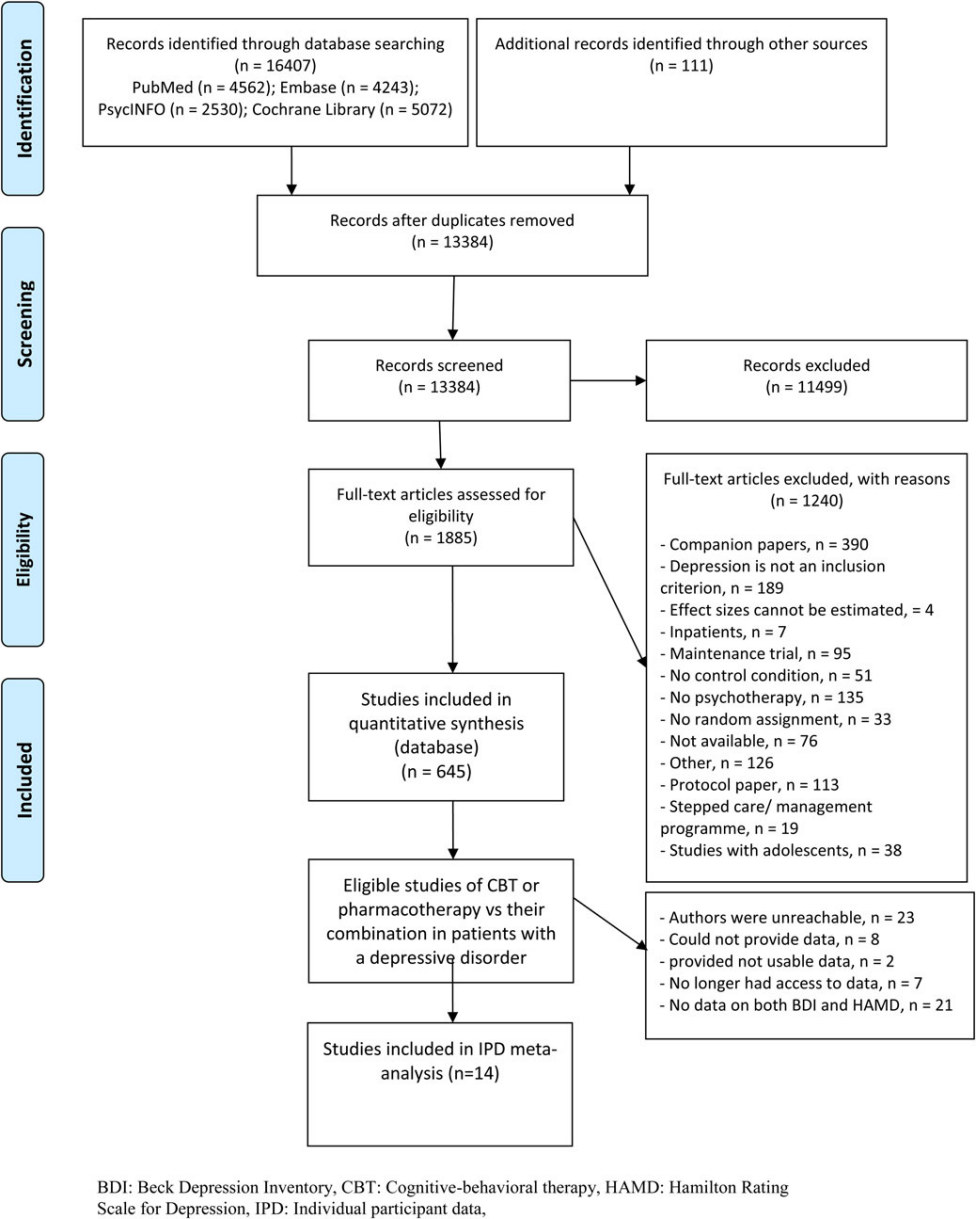
Flowchart of study identification.

Figure 1 presents the flow of the literature search. For this study we used the search that was conducted in January 2016. After removing the duplicates from different data sources, two independent reviewers examined 13 384 titles and abstracts, retrieved 1885 full-text articles and finally identified 75 studies that compared CBT, antidepressant pharmacotherapy or their combination in the acute phase treatment of depression.

Of these, authors of 14 studies provided IPD including both HAMD and BDI (Rush *et al*., 1977; Murphy *et al*., 1984; Elkin *et al*., 1989; Hollon *et al*., 1992; Jarrett *et al*., 1999; Reynolds *et al*., 1999; Mohr *et al*., 2001) or BDI-II (DeRubeis *et al*., 2005; Dimidjian *et al*., 2006; Lesperance *et al*., 2007; McBride *et al*., 2007; Dozois *et al*., 2009; Hegerl *et al*., 2010; Quilty *et al*., 2014) (Table 1). Studies using the BDI were published mainly before 2000, while those using the BDI-II were all published after 2000. The 14 studies included 1536 participants: their mean age was around 40 years, and 61% were women. The treatment lasted between 10 and 24 weeks, typically for 16 weeks. At baseline, participants presented with average HAMD scores around 20, which dropped to scores around 9 at end of treatment. Seven studies used the BDI, which dropped from around 26 to around 10; another seven studies used the BDI-II, which dropped from around 30 to 12, on average.

**Table 1.** Included studies and their characteristics

Studies	*N*	Age	Sex (male/female)	Treatments	Treatment duration (weeks)	Scale used for inclusion	Baseline		Endpoint	
Using BDI							HAMD	BDI	HAMD	BDI
Elkin *et al*. (1989)	116	34.5	35/81	CBT *v.* ADM	16	HAMD ⩾ 14	19.5 (4.2)	26.7 (8.5)	7.8 (6.8)	9.0 (9.7)
Hollon *et al*. (1992)	107	32.6	21/86	CBT *v.* ADM *v.* CBT + ADM	12	HAMD ⩾ 14 and BDI ⩾ 20	23.9 (4.9)	30.7 (7.0)	7.7 (8.2)	9.4 (9.9)
Jarrett *et al*. (1999)	72	39.3	21/51	CBT *v.* ADM	10	HAMD ⩾ 14	16.6 (3.1)	25.3 (7.9)	8.1 (6.6)	8.6 (7.8)
Mohr *et al*. (2001)	45	43.9	9/35	CBT *v.* ADM	16	HAMD ⩾ 16 and BDI ⩾ 16	19.5 (3.8)	20.7 (5.3)	13.4 (6.5)	13.5 (9.5)
Murphy *et al*. (1984)	33	–	6/27	CBT *v.* ADM	12	HAMD ⩾ 14 and BDI ⩾ 20	18.9 (2.9)	28.9 (6.2)	6.0 (5.1)	8.1 (8.5)
Reynolds *et al*. (1999)	58	67.3	12/46	IPT *v.* ADM *v.* IPT + ADM	16	SADS-L and RDC and SCID	19.7 (4.1)	17.7 (8.0)	12.4 (4.1)	11.6 (8.1)
Rush *et al*. (1977)	41	–	15/26	CBT *v.* ADM	12	HAMD ⩾ 14 and BDI ⩾ 20	21.3 (4.0)	30.2 (6.1)	7.3 (5.4)	9.2 (9.8)
*All patients with BDI*	*472*	*40.5*	*117/350*				*20.2 (4.7)*	*26.2 (8.5)*	*8.9 (6.9)*	*9.8 (9.2)*
Using BDI-II							HAMD	BDI-II	HAMD	BDI-II
DeRubeis *et al*. (2005)	180	39.9	75/105	CBT *v.* ADM	16	HAMD ⩾ 20	21.5 (4.0)	32.6 (9.4)	8.3 (6.3)	10.3 (10.4)
Dimidjian *et al*. (2006)	145	38.9	44/101	CBT *v.* ADM	16	HAMD ⩾ 14 and BDI-II ⩾ 20	18.5 (4.1)	31.9 (7.4)	7.1 (5.6)	9.9 (10.5)
Dozois *et al*. (2009)	48	45.3	12/36	ADM *v.* CBT + ADM	15	SCID	18.0 (4.0)	28.6 (9.9)	7.0 (6.6)	12.6 (11.3)
Hegerl *et al*. (2010)	144	46.7	47/97	CBT *v.* ADM	10	HAMD ⩾ 8 ⩽ 22	16.5 (4.3)	21.1 (8.3)	9.0 (6.7)	11.2 (8.3)
Lesperance *et al*. (2007)	142	57.9	109/33	ADM *v.* IPT + ADM	12	HAMD ⩾ 20	22.3 (4.9)	30.3 (9.1)	11.7 (7.9)	15.4 (11.1)
McBride *et al*. (2007)	301	37.4	133/168	CBT *v.* ADM	24	SCID	19.3 (3.7)	31.9 (9.2)	6.6 (4.9)	11.4 (10.7)
Quilty *et al*. (2014)	104	33.5	50/54	CBT *v.* ADM	16	SCID	16.6 (5.1)	29.8 (8.6)	7.9 (6.2)	12.0 (10.8)
*All patients with BDI-II*	*1064*	*42.0*	*447/527*				*19.3 (4.7)*	*30.4 (9.4)*	*8.5 (6.5)*	*12.0 (10.6)*

ADM, antidepressant medication; BDI, Beck Depression Inventory; CBT, cognitive-behavioural therapy; HAMD, Hamilton Rating Scale for Depression; IPT, interpersonal psychotherapy; RDC, Research Diagnostic Criteria; SADS-L, Schedule for Affective Disorders and Schizophrenia-Lifetime Version; SCID, Structured Interview for DSM.

Standard deviations in parentheses.

### Correlations between HAMD and BDI, BDI-II

**Fig. 2.**
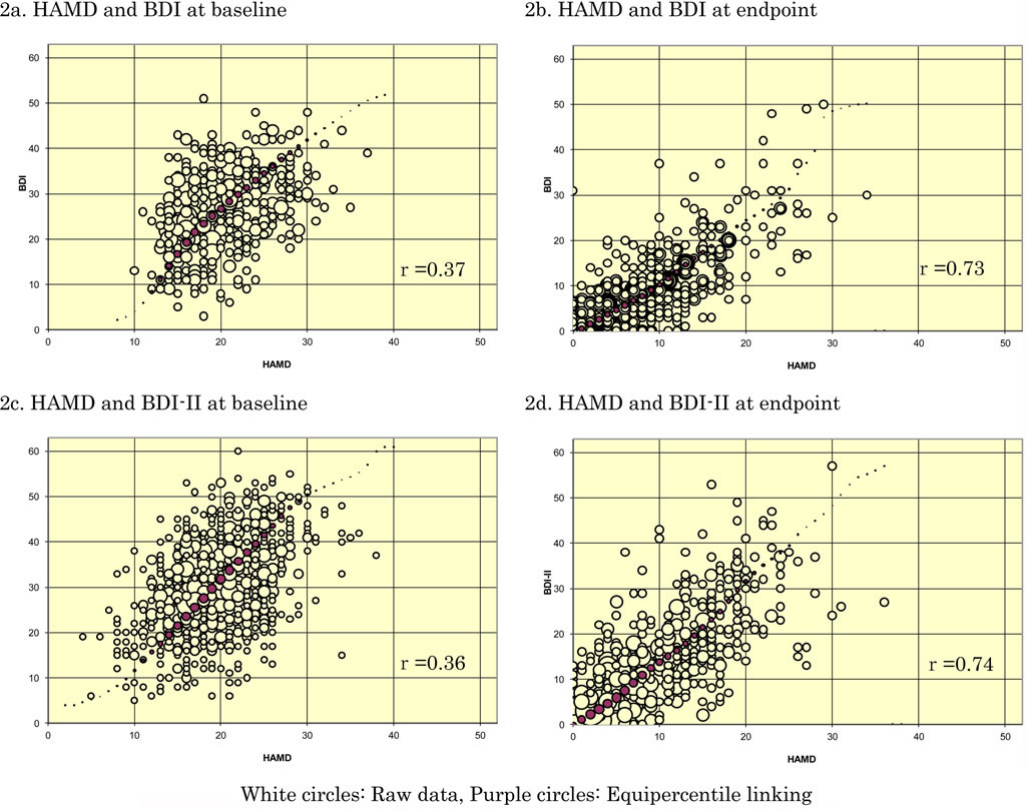
Scatterplots of HAMD and BDI, BDI-II, superimposed with equipercentile linking.

Figure 2 presents the scatterplots between HAMD and BDI or BDI-II at baseline and at end of treatment. The correlations between the HAMD and BDI or BDI-II were relatively weak, with Spearman correlation coefficients of 0.37 and 0.36, respectively: the raw data were scattered relatively widely, and there were few data points with a HAMD score of 10 or below, or 30 or higher. At the end of treatment, the correlations between the HAMD and BDI or BDI-II were stronger (*r* = 0.73 and 0.74, respectively), with raw data distributed in a more elliptic manner predominantly below a HAMD score of 20. When the baseline observations and end-of-treatment observations were combined, the correlations between the scales rose to 0.77 and 0.76, respectively.

There were moderately strong correlations between change scores: the Spearman correlation coefficients were 0.69 and 0.61 for the HAMD and the BDI or BDI-II change scores, respectively.

### Subgroup and sensitivity analyses

Appendix B shows the scatterplots for men and women separately at baseline and at endpoint. Appendix C provides the scatterplots for those who would later drop out and those who would complete the studies separately. The equipercentile linkings were essentially similar across these subgroups, and hence with the overall findings.

### Linking HAMD and BDI, BDI-II

**Fig. 3.**
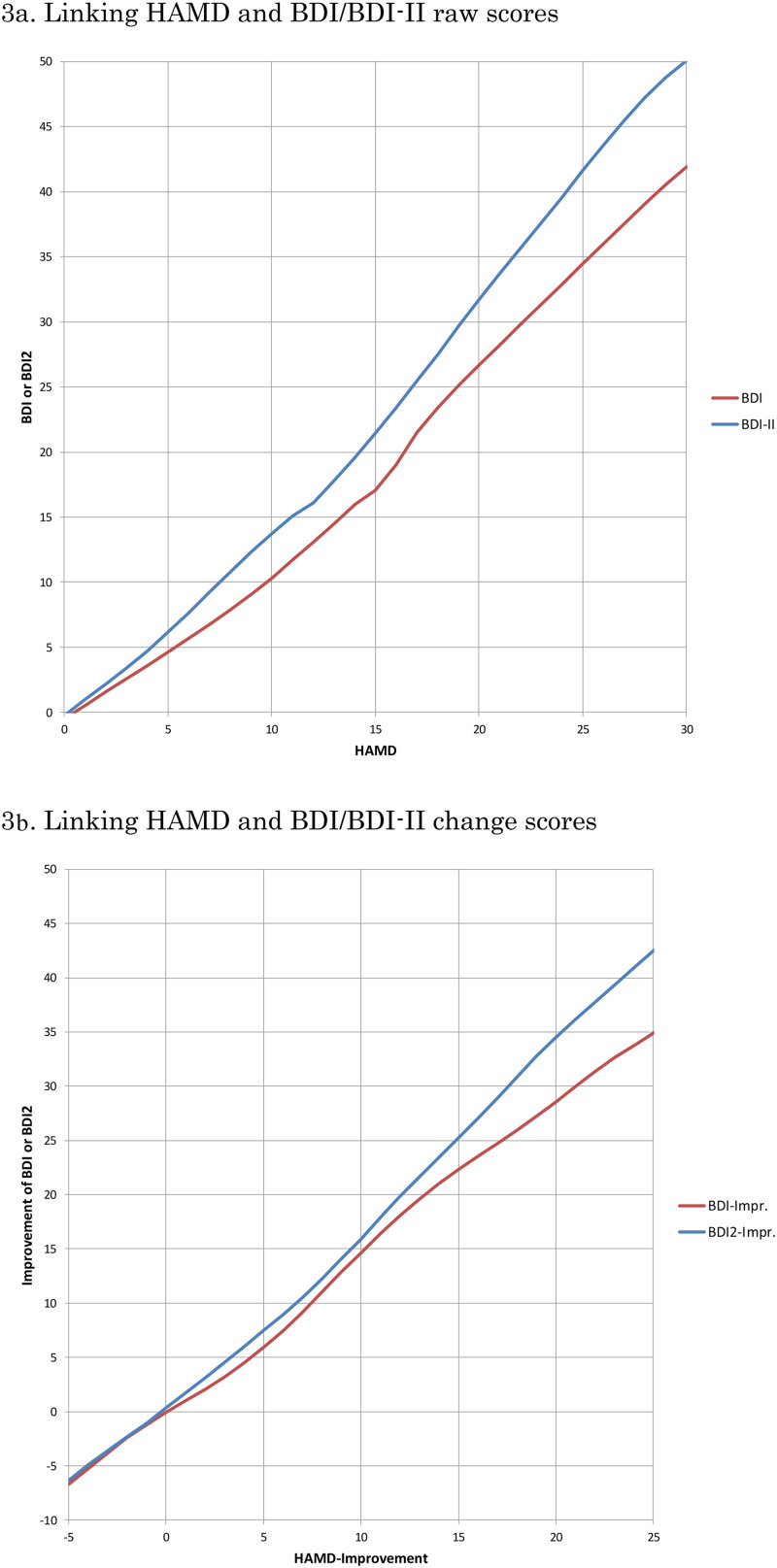
Linking curves between HAMD and BDI, BDI-II.

Figure 3 depicts the linking curves between HAMD and BDI or BDI-II: 3a in terms of raw scores and 3b in terms of change scores. Table 2 summarises the correspondences on each of these scales. Outside of the ranges displayed and tabled; there were too few data for linking.

**Table 2.** Conversion from HAMD to BDI or BDI-II scores

Total scores			Change scores		
HAMD	BDI	BDI-II	HAMD	BDI	BDI-II
0	0	0	−5	−7	−6
1	1	1	−4	−5	−5
2	2	2	−3	−4	−4
3	3	3	−2	−2	−2
4	4	5	−1	−1	−1
5	5	6	0	0	0
6	6	8	1	1	2
7	7	9	2	2	3
8	8	11	3	3	5
9	9	12	4	4	6
10	10	13	5	6	7
11	12	15	6	7	9
12	13	16	7	9	11
13	15	18	8	11	12
14	16	20	9	13	14
15	17	21	10	15	16
16	19	23	11	16	18
17	21	25	12	18	20
18	23	27	13	20	22
19	25	30	14	21	23
20	27	32	15	22	25
21	28	34	16	24	27
22	30	36	17	25	29
23	31	38	18	26	31
24	33	40	19	27	33
25	34	42	20	29	35
26	36	44	21	30	36
27	38	45	22	31	38
28	39	47	23	33	39
29	41	49	24	34	41
30	42	50	25	35	43

BDI, Beck Depression Inventory; BDI-II, Beck Depression Inventory, 2nd Edition; HAMD, Hamilton Rating Scale for Depression.

## Discussion

We have obtained IPD from 14 randomised controlled trials of psychotherapies for the acute phase treatment of depression (total *n* = 1536 participants), in which the HAMD and the BDI/BDI-II were administered concurrently both at baseline and at end of treatment. The equipercentile linking between the HAMD and the BDI/BDI-II raw scores or change scores established that the HAMD scores of 10, 20 and 30 corresponded approximately with the BDI of 10, 27 and 42 or with the BDI-II of 13, 32 and 50; the HAMD change scores of −20 and −10 with the BDI of −29 and −15 and with the BDI-II of −35 and −16.

It is worthwhile to note that the BDI-II tended to produce higher scores than the original BDI. This was noted originally when the BDI-II was first developed (Beck *et al*., 1996) and replicated subsequently (Dozois *et al*., 1998), as the BDI-II dropped or reworded items that poorly reflected depression severity in the original BDI. Our linking analyses correctly reflected this difference between the BDI and the BDI-II.

Possible weaknesses of this study include the following. First, our IPD dataset included only trials that compared psychotherapies against pharmacotherapies or their combinations. Some might suspect that the relationship between the HAMD and the BDI/BDI-II could be different if the data were derived from pharmacotherapy trials. Likewise, the datasets were limited to individuals with major depression who sought treatment. It is possible that linking results could be different among people in the community suffering from major depression but not seeking treatment. However, as this linking study is not about treatments but about measurements, we do not foresee any strong reason that there would be major differences. Second, the correlations between the HAMD and the BDI/BDI-II were only moderate at baseline. This is reflected by rounder, rather than elliptic, scatterplots between the HAMD and the BDI/BDI-II at baseline (Fig. 2). We originally suspected that there may have arisen some ‘baseline inflation’ through which people tended to overestimate the depression severity at baseline when a certain threshold on that scale was used as a cutoff criterion for eligibility. Focusing on the four studies that did not use a cutoff (cf Table 1), however, did not improve the correlation coefficients at baseline. A possibility remains that the observed low correlation at baseline is due to range restriction of the available scores on HAMD and BDI/BDI-II as indicated by smaller standard deviations of these scores at baseline than at endpoint. Another possibility is that participants may have been engaging in impression management, either by overreporting or underreporting their symptoms in the self-reports, especially at the start of the trial: as the trial progresses, they may feel less need for such impression management. Third, it must be pointed out that observer- and self-ratings of depression severity do not in general show perfect correlations and that their contrasts can sometimes provide clinically useful information (Petkova *et al*., 2000; Targum *et al*., 2013). The conversion algorithm as presented in this study must therefore serve as a rough guide when only one of HAMD/BDI/BDI-II is available and one wishes to know the approximately equivalent scores. Last, the linking above the HAMD scores of 30, where there were few endpoint measurements, may require appropriate caution. Alternatively it may be safer to convert the change scores rather than raw scores when researchers would like to use one common scale across different studies.

On the other hand, the current study also has several major strengths. This is the first study to empirically link the most representative observer-rated instrument and the most frequently used self-rating instrument for depression, based on data from over 1500 participants. The conversion table will help clinicians interpret the HAMD or BDI/BDI-II scores of their patients in a more versatile manner as they can now convert each scale into another. Clinicians will also find it easier to compare their patients’ scores with those reported in the literature when the latter only reports one of these scales while they have only scores from the other scales for their patients. The conversion table will also be informative for researchers when they compare trials using one but not the other of these scales; in particular, the table will allow researchers to convert these scales onto the common scale so that they would need less assumption when they conduct IPD meta-analysis (Furukawa *et al*., 2018); without the conversion the only way to pool individual data was via standardisation assuming a consistent and common standard deviation (Bower *et al*., 2013). For the latter purpose one might prefer to use the conversion of the change scores as they showed higher correlations among the scales.

In conclusion, this study provided the first empirically-derived conversion table between the HAMD and the BDI/BDI-II. The table is expected to be of help to both clinicians and researchers.

## Data and materials

Data used in this study are not available for sharing due to ethical and data management requirements. The researchers are open to collaboration.

## Appendix

Translating the BDI and BDI-II into the HAMD and vice versa with equipercentile linking

### Appendix A. Search string for PubMed

(Psychotherapy [MH] OR psychotherap*[All Fields] OR cbt[All Fields] OR “behavior therapies”[All Fields] OR “behavior therapy”[All Fields] OR “behavior therapeutic”[All Fields] OR “behavior therapeutical”[All Fields] OR “behavior therapeutics”[All Fields] OR “behavior therapeutist”[all Fields] OR “behavior therapeutists”[All Fields] OR “behavior treatment”[All Fields] OR “behavior treatments”[All Fields] OR “behaviors therapies”[All Fields] OR “behaviors therapy”[All Fields] OR “behaviors therapeutics”[All Fields] OR “behaviors therapeutic”[All Fields] OR “behaviors therapeutical”[All Fields] OR “behaviors therapeutist”[All Fields] OR “behaviors therapeutists”[All Fields] OR “behaviors treatment”[All Fields] OR “behaviors treatments”[All Fields] OR “behavioral therapies”[All Fields] OR “behavioral therapy”[All Fields] OR “behavioral therapeutics”[All Fields] OR “behavioral therapeutic”[All Fields] OR “behavioral therapeutical”[All Fields] OR “behavioral therapeutist”[All Fields] OR “behavioral therapeutists”[All Fields] OR “behavioral treatment”[All Fields] OR “behavioral treatments”[All Fields] OR “behaviour therapies”[All Fields] OR “behaviour therapy”[All Fields] OR “behaviour therapeutic”[All Fields] OR “behaviour therapeutical”[All Fields] OR “behaviour therapeutics”[All Fields] OR “behaviour therapeutist”[all Fields] OR “behaviour therapeutists”[All Fields] OR “behaviour treatment”[All Fields] OR “behaviour treatments”[All Fields] OR “behaviours therapies”[All Fields] OR “behaviours therapy”[All Fields] OR “behaviours therapeutics”[All Fields] OR “behaviours therapeutic”[All Fields] OR “behaviours therapeutical”[All Fields] OR “behaviours therapeutist”[All Fields] OR “behaviours therapeutists”[All Fields] OR “behaviours treatment”[All Fields] OR “behaviours treatments”[All Fields] OR “behavioural therapies” [All Fields] OR “behavioural therapy”[All Fields] OR “behavioural therapeutics”[All Fields] OR “behavioural therapeutic”[All Fields] OR “behavioural therapeutical”[All Fields] OR “behavioural therapeutist”[All Fields] OR “behavioural therapeutists”[All Fields] OR “behavioural treatment”[All Fields] OR “behavioural treatments”[All Fields] OR “cognition therapies”[All Fields] OR “cognition therapie”[All Fields] OR “cognition therapy”[All Fields] OR “cognition therapeutical”[All Fields] OR “cognition therapeutic”[All Fields] OR “cognition therapeutics”[All Fields] OR “cognition therapeutist”[All Fields] OR “cognition therapeutists”[All Fields] OR “cognition treatment”[All Fields] OR “cognition treatments”[All Fields] OR psychodynamic[All Fields] OR Psychoanalysis[MH] OR psychoanalysis[All Fields] OR psychoanalytic*[All Fields] OR counselling[All Fields] OR counseling[All Fields] OR Counseling[MH] OR “problem-solving”[All Fields] OR mindfulness[All Fields] OR (acceptance[All Fields] AND commitment[All Fields]) OR “assertiveness training”[All Fields] OR “behavior activation”[All Fields] OR “behaviors activation”[All Fields] OR “behavioral activation”[All Fields] OR “cognitive therapies”[All Fields] OR “cognitive therapy”[All Fields] OR “cognitive therapeutic”[All Fields] OR “cognitive therapeutics”[All Fields] OR “cognitive therapeutical”[All Fields] OR “cognitive therapeutist”[All Fields] OR “cognitive therapeutists”[All Fields] OR “cognitive treatment”[All Fields] OR “cognitive treatments”[All Fields] OR “cognitive restructuring”[All Fields] OR ((“compassion-focused”[All Fields] OR “compassion-focussed”[All Fields]) AND (therapy[SH] OR therapies[All Fields] OR therapy[All Fields] OR therape*[All Fields] OR therapis*[All Fields]OR Therapeutics [OR treatment*[All Fields])) OR ((therapy[SH] OR therapies[All Fields]

OR therapy [All Fields] OR therape*[All Fields] OR therapis*[All Fields] OR Therapeutics[MH] OR treatment*[All Fields]) AND constructivist*[All Fields]) OR “metacognitive therapies”[All Fields] OR “metacognitive therapy”[All Fields] OR “metacognitive therapeutic”[All Fields] OR “metacognitive therapeutics”[All Fields] OR “metacognitive therapeutical”[All Fields] OR “metacognitive therapeutist”[All Fields] OR “metacognitive therapeutists”[All Fields] OR “metacognitive treatment”[All Fields] OR “metacognitive treatments”[All Fields] OR “meta-cognitive therapies”[All Fields] OR “meta-cognitive therapy”[All Fields] OR “meta-cognitive therapeutic”[All Fields] OR “meta-cognitive therapeutics”[All Fields] OR “meta-cognitive therapeutical”[All Fields] OR “meta-cognitive therapeutist”[All Fields] OR “meta-cognitive therapeutists”[All Fields] OR “meta-cognitive treatment”[All Fields] OR “meta-cognitive treatments”[All Fields] OR “solution-focused therapies”[All Fields] OR “solution-focused therapy”[All Fields] OR “solution-focused therapeutic”[All Fields] OR “solution-focused therapeutics” [All Fields] OR “solution-focused therapeutical”[All Fields] OR “solution focused therapies”[All Fields] OR “solution focused therapy”[All Fields] OR “solution focused therapeutic”[All Fields] OR “solution focused therapeutics”[All Fields] OR “solution focused therapeutical”[All Fields]OR “solution-focussed therapies”[All Fields] OR “solution-focussed therapy”[All Fields] OR “solution-focussed therapeutic”[All Fields] OR “solution-focussed therapeutics”[All Fields] OR “solution-focussed therapeutical”[All Fields]OR “solution focussed therapies”[All Fields] OR “solution focussed therapy”[All Fields] OR “solution focussed therapeutic”[All Fields] OR “solution focussed therapeutics”[All Fields] OR “solution focussed therapeutical”[All Fields] OR “self-control therapies”[All Fields] OR “self-control therapy”[All Fields] OR “self-control therapeutics”[All Fields] OR “self-control therapeutical”[All Fields] OR “self-control therapeutic”[All Fields] OR “self-control training”[All Fields] OR “self-control trainings”[All Fields] OR “self control therapies”[All Fields] OR “self control therapy”[All Fields] OR “self control therapeutics”[All Fields] OR “self control therapeutical”[All Fields] OR “self control therapeutic”[All Fields] OR “self control training”[All Fields] OR “self control trainings”[All Fields] AND (Depressive Disorder[MH] OR Depression[MH]OR dysthymi*[All Fields] OR “affective disorder”[All Fields]OR “affective disorders”[All Fields] OR “mood disorder”[All Fields] OR “mood disorders”[All Fields] OR depression*[All Fields] OR depressive*[All Fields] OR “dysthymic disorder”[MeSH Terms]) AND ((randomized controlled trial [pt] OR controlled clinical trial [pt] OR randomized [tiab] OR randomly [tiab] NOT (animals[mh] NOT (animals[mh] AND humans [mh]))

### Appendix B. Subgroup analyses by sex

B1. Men

B2. Women

### Appendix C. Subgroup analyses by completers and dropouts

C1. Dropouts

C2. Completers
